# Alcohol and male infertility: is there a safe dose? Evidence of accessory gland dysfunction, oxidative stress, and sperm DNA damage

**DOI:** 10.3389/frph.2026.1934203

**Published:** 2026-09-29

**Authors:** P. Jagadesh, T. B. Sridharan

**Affiliations:** 1 Gene Cloning Technology Lab, School of Biosciences and Technology, Vellore Institute of Technology, Vellore, India; 2 VIT Bhopal University, Bhopal, India

**Keywords:** alcohol consumption, DNA damage, lifestyle and behavior, male infertility (primary and secondary) accessory glands, reproductive health, sperm quality

## Abstract

**Introduction:**

Alcohol consumption is a recognized modifiable lifestyle factor affecting male reproductive health. Yet, its dose-dependent effects on accessory gland function and sperm genomic integrity remains poorly elucidated. This cross-sectional study examined the relationship between alcohol consumption and the accessory gland-sperm function axis, focusing on the mediating role of oxidative stress in cellular and molecular damage.

**Methods:**

A total of 126 men undergoing fertility evaluation were stratified into a control cohort (non–drinkers with normozoospermia, *n* = 25) and three alcohol–consuming infertile cohorts: mild (*n* = 37), moderate (*n* = 31), and heavy (*n* = 33). A standard semen analysis was performed following world Health Organization (2021) guidelines, while seminal plasma was quantified for accessory gland markers (fructose and total protein concentration) and an oxidative stress marker (malondialdehyde levels). Nuclear maturity and DNA integrity were evaluated using aniline blue and acridine orange fluorescence staining.

**Results:**

The findings demonstrated that alcohol consumption was associated with a dose-dependent deterioration across all standard semen parameters, particularly correlating with teratozoospermia (<4% normal morphology). Seminal fructose and total protein concentrations were decreased significantly with alcohol intake levels (*p* < 0.0001), whereas MDA levels exhibited a drastic elevation (*p* < 0.0001). A correlation analysis revealed a strong positive association between seminal fructose and total motility (r = 0.867, *p* < 0.0001). Furthermore, MDA levels were strongly correlated with chromatin condensation defects (r = 0.841) and the sperm DNA fragmentation index (r = 0.880, *p* < 0.0001).

**Discussion:**

These findings suggest that chronic alcohol consumption is associated with impaired male fertility potential by supressing accessory gland secretory function and with elevated systemic oxidative stress. Even long-term mild alcohol consumption was associated with considerable levels of oxidative stress that affect chromatin packaging and DNA integrity. Men undergoing fertility evaluation, particularly prior to assisted reproductive techniques, may benefit from counseling on alcohol reduction or abstinence to safeguard embryo genomic integrity.

## 1 Introduction

Infertility is defined as the inability of a couple to achieve pregnancy even after 12 months of regular unprotected sexual intercourse. Infertility is considered one of the serious global health concerns affecting millions of couples worldwide, in which almost 50% involve male factor–mediated infertility (1). The etiology of male infertility is often complex, as it is multifactorial, with almost half of the cases being idiopathic (2–4). In recent decades, there has been a major focus on the role of modifiable lifestyle factors, which include diet, stress, smoking, substance use, and alcohol consumption, as a key contributor to the deterioration of male reproductive health (2, 5).

Alcohol consumption is common nowadays, and approximately 44% of the global population is reported to consume alcohol, with an increased proportion of males (6). It is one of the most widely consumed psychoactive substances with numerous health effects in humans, and its impact on male fertility remains a subject of considerable debate (7). The primary mechanism is the induction of testicular toxicity via oxidative stress. The ethanol-induced testicular toxicity is primarily driven by the oxidative metabolism of ethanol within the testicular tissues (8). Although the liver is the primary site of metabolism, the testicular tissue expresses both alcohol dehydrogenase and cytochrome P450-2E1 (CYP2E1). Chronic ethanol exposure induces the upregulation of CYP2E1, which metabolizes the ethanol to acetaldehyde, leading to the generation of free radicals like superoxide anions and hydroxyl radicals, inducing oxidative stress (9). These elevated levels of reactive oxygen species (ROS) overwhelm the testicular antioxidant defense system. Consequently, these free radicals interact with the polyunsaturated fatty acids in the sperm cell membrane, enhancing the lipid peroxidation cascades that compromise membrane integrity and trigger germ cell apoptosis via the intrinsic mitochondrial pathway (10).

Several research studies and meta-analyses have linked chronic or heavy alcohol consumption to detrimental effects on human semen quality parameters (11–13). However, this association is not universally consistent. Several other studies have reported that moderate alcohol consumption is positively associated with semen quality (14). These contrary studies highlight a critical need for studies that employ a precise dose–response categorization to identify the alcohol-induced reproductive toxicity.

Alcohol may also compromise the integrity of male accessory gland function. Seminal plasma is a complex fluid rich in proteins and fructose that are vital for sperm survival and function. The seminal vesicle secretes fructose, which acts as a metabolic fuel for sperm motility and may serve as a clinical marker for seminal vesicle function (15). Total protein concentration (TPC) reflects the secretory activity of the prostate and vesicles (16). It plays a crucial role in preventing sperm cells from immune rejection when interacting with the female reproductive tract and can serve as biomarkers for male reproductive health (17). The specific impact of alcohol on the accessory gland function remains poorly stratified in dose-dependent studies.

Excess oxidative stress leads to intracellular redox imbalance by elevating oxidative stress markers like malondialdehyde (MDA) as a result of sperm membrane lipid peroxidation (18). Beyond sperm membrane lipid peroxidation, the effects of oxidative stress extend to sperm DNA and chromatin condensation, which are critical predictors of fertilization potential and embryo development. Ethanol-induced oxidative stress can disrupt the critical histone-to-protamine transition during spermiogenesis through the generation of reactive aldehydes, specifically 4-hydroxynonenal (4-HNE), which is a product of lipid peroxidation. 4-HNE effectively blocks the formation of essential disulfide bridges because of its electrophilic nature; it forms covalent adducts with these nucleophilic thiol (–SH) groups on protamine, which leads to poor chromatin condensation, rendering the sperm nucleus physically unstable and highly susceptible to further oxidative attack and DNA fragmentation (19). Despite these established biological mechanisms, a critical research gap exists. Only a few human studies have been done on the effects of alcohol consumption on human male infertility. Therefore, the aim of this study was to investigate the dose–response relationship between alcohol consumption and semen quality and to elucidate the potential mechanistic role of oxidative stress and accessory gland dysfunction in mediating these effects. Understanding these impacts can help couples undergoing fertility treatments, such as *in vitro* fertilization (IVF), optimize treatment procedures and raise success rates.

Although the deleterious effects of alcohol on spermatogenesis are recognized, the existing literature often treats alcohol consumption as a binary variable (drinker vs. non-drinker) and focuses predominantly on semen parameter endpoints. Our study reveals a mechanistic insight into how alcohol consumption systematically deteriorates male fertility potential at both cellular and molecular levels.

## 2 Methods

### 2.1 Ethics approval and study design

All procedures were performed in compliance with relevant laws and institutional guidelines and have been approved by the Institutional Ethical Committee of Sri Narayani Hospital & Research Centre (SNHRC), Vellore (Ref. No.: SNHRCIEC/67/29/03/25) and Institutional Human Ethical Committee of Vellore Institute of Technology (VIT University), Vellore (Ref. No.: VIT/IECH/2025/18 IECH/8 November 2025/12). The nature and purpose of the study were verbally explained to all participants prior to enrollment. Each participant was provided with a printed document outlining the planned analytical procedures, and written consent was obtained. This process ensured explicit authorization for specific downstream evaluations, including scanning electron microscopy (SEM), chromatin condensation assays, and DNA fragmentation analysis. To ensure strict patient confidentiality, all self-reported histories, demographic data, and biological samples were de-identified and assigned unique numeric codes immediately upon collection.

This study included individuals with a history of alcohol consumption for a minimum of 1 year with no other substance use, such as tobacco or any other recreational drugs, to isolate the specific toxicological impact of alcohol. Healthy non-drinkers or strict abstainers with normozoospermia were considered the control group. Patients with a habit of smoking, chronic illness, varicocele, history of cancer, reproductive or other surgeries impacting fertility, and systemic infections and those who were undergoing medications or therapies impacting fertility were excluded from the study.

Alcohol consumption patterns were assessed using a structured questionnaire capturing the frequency, type, and volume of beverage intake over the preceding year. Alcohol intake was then converted into standard drink units, where one standard drink is approximately 14 g of pure ethanol. Based on these calculated volumes, participants were stratified into three cohorts: Mild, Moderate, and Heavy, with cutoffs adapted from the low-risk and heavy-drinking thresholds defined by the NIAAA: mild (≤3 standard drinks per week), moderate (4–14 standard drinks per week), and heavy (≥15 standard drinks per week or ˃4 drinks on any single day). The stratification thresholds defining the moderate and heavy groups align with the NIAAA's established low-risk and heavy-drinking limits for men; the intermediate Mild category was defined to allow finer dose stratification within the low-risk range (29).

The study groups were classified as four groups: Subjects with normozoospermia and no history of alcohol consumption were included in the control group. Based on self-reported alcohol intake, the infertile patient population was classified into three experimental cohorts: mild, moderate, and heavy (Figure 1).

**Figure 1:**
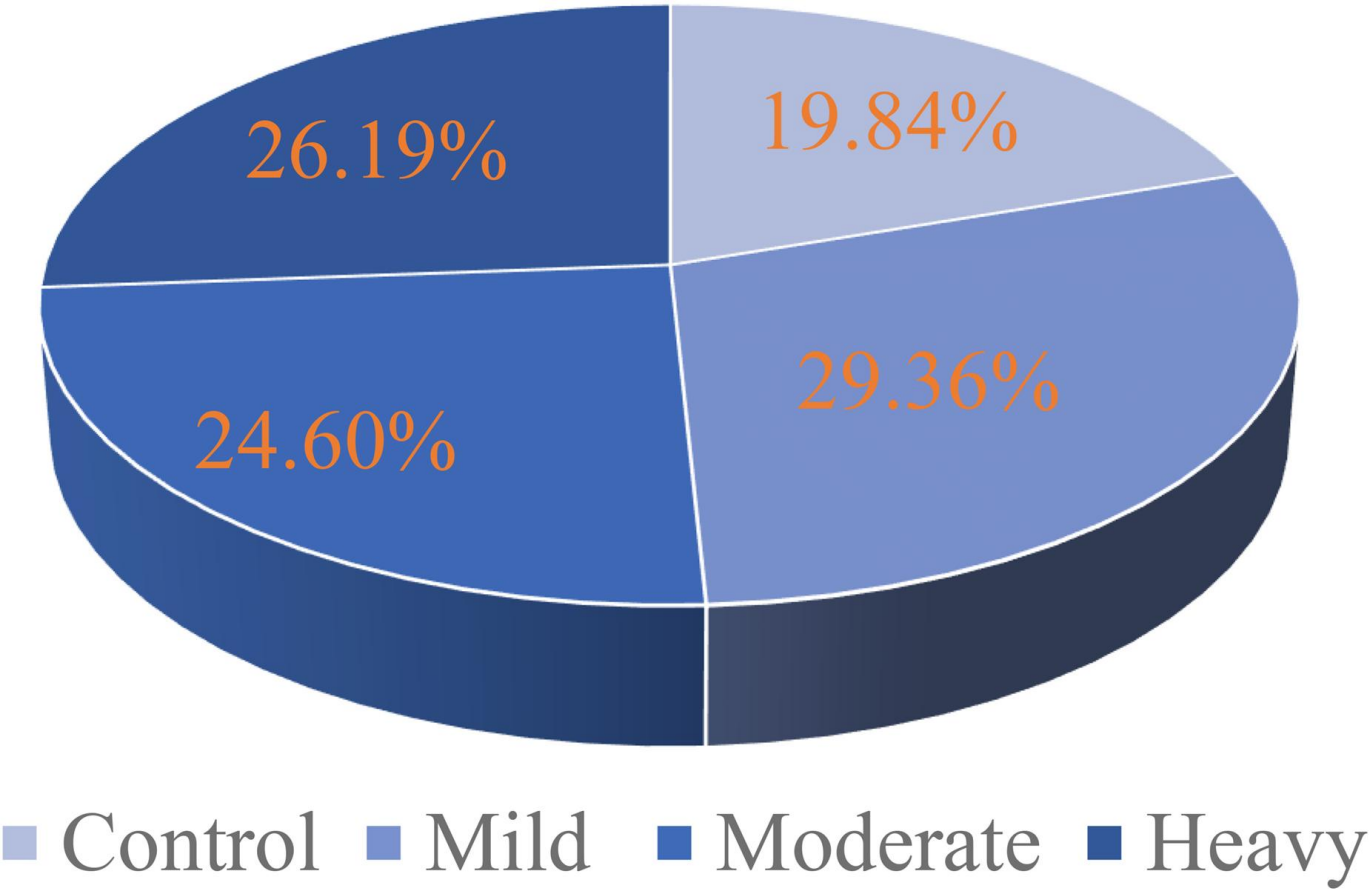
A pie chart illustrates the stratification of the total study population (*n* = 126). The control group (*n* = 25) comprised normozoospermia subjects with no history of alcohol consumption. Based on self-reported intake, the alcohol-consuming population was classified into three experimental cohorts: mild (*n* = 37), moderate (*n* = 31), and heavy (*n* = 33).

### 2.2 Sample collection and analysis

Samples were collected by masturbation in a sterile, wide-mouthed plastic container following 3–5 days of abstinence. The collected semen samples were allowed to liquefy at 37 °C for 30 min and analyzed according to the sixth edition of the World Health Organization (WHO) Laboratory Manual for the Examination and Processing of Human Semen (20). Sperm count, concentration, and motility were assessed using a Makler's reusable counting chamber. A 10 μL aliquot of thoroughly mixed, undiluted liquefied semen was placed in the center of the chamber and covered with optical cover glass. The sample was examined under a phase-contrast microscope at 200× magnification. The number of spermatozoa counted in any strip of 10 squares indicated the concentration in millions per milliliter (10^6^/mL).

The sperm morphology was evaluated by following the strict Kruger criteria. A 4% or more normal form (oval head with a well-defined acrosome, distinct midpiece, and tail) was considered an acceptable range for a man to be fertile according to WHO criteria. These criteria help predict fertility potential, often leading to IVF or ICSI.

#### 2.2.1 Morphology analysis

For qualitative visual confirmation of structural defects, the semen samples were processed for a SEM analysis. Sperm cells were separated by centrifugation at 1,500 rpm for 10 min and washed twice in phosphate buffered saline (PBS, pH 7.4) to remove debris. The pellet was then coated on a coverslip and fixed in 2.5% glutaraldehyde for 2 h at 4 °C. Following fixation, the samples were washed in PBS and subjected to graded dehydration using an ascending ethanol series (30%, 50%, 70%, 90%, and 100% absolute alcohol) for 10 min each. To remove residual moisture without damaging the membrane structure, the samples were critical point-dried using CO_₂_. The dried samples were mounted on aluminum stubs and sputter-coated with a thin layer of gold palladium to enhance conductivity (21). The specimen was examined under a scanning electron microscope (CARL ZEISS, Model: EVO 18 Research).

### 2.3 Biochemical analysis of accessory gland function

#### 2.3.1 Fructose concentration

Seminal fructose concentration was determined by using the resorcinol method (22). A quantity of 100 μL of seminal plasma was mixed with 200 μL of sodium hydroxide and 200 μL of zinc sulfate to deproteinize it. Then, it was centrifuged at 3,000*g* for 10 min to obtain a protein-free supernatant. A quantity of 200 μL of the supernatant was mixed with 200 μL of 0.1% resorcinol and 660 μL of 10 N HCl. Then, the reaction mixture was kept in a hot water bath at 90 °C for 15 min. After cooling to room temperature, the absorbance was measured at 490 nm using a UV spectrophotometer. The obtained values were spotted against the standard curve prepared by using D-fructose (Sigma-Aldrich).

#### 2.3.2 Total protein concentration

The TPC in the seminal plasma was analyzed by following the Lowry's protocol (23). A 250 μL aliquot of seminal plasma were mixed with 2.5 mL of alkaline copper reagent (2% Na_2_CO_3_​ in 0.1 N NaOH with 0.5% CuSO_4_​·5H_2_O in 1% sodium potassium tartrate in a 50:1 ratio). The mixture was mixed thoroughly and incubated at room temperature for 10 min. Then, 0.25 mL of the Folin–Ciocalteu reagent (diluted 1:1 with distilled water) was added and incubated in the dark for 30 min. Using a spectrophotometer, absorbance was measured at 660 nm. Bovine serum albumin was used as a standard.

### 2.4 Oxidative stress markers

#### 2.4.1 MDA analysis

A thiobarbituric acid (TBA) assay was used to measure lipid peroxidation in seminal plasma by measuring MDA concentration (24). A quantity of 100 μL of seminal plasma was mixed with 900 μL of the TBA reagent (0.8% TBA, 0.25 N HCl, and 10% trichloroacetic acid). The mixture was incubated in a boiling water bath at 95 °C for 60 min to allow the formation of the MDA–TBA adduct. Then, samples were cooled to room temperature and centrifuged at 1,000×*g* for 10 min. Absorbance of the supernatant was measured spectrophotometrically at 534 nm. Tetraethoxypropane was used as a standard.

### 2.5 Assessment of sperm membrane and functional integrity

#### 2.5.1 Hypo-osmotic swelling (HOS) test

To assess the functional integrity of the sperm cell plasma membrane, the HOS test was performed based on WHO guidelines (20). A 10 µL semen sample was mixed with 100 µL of hypo-osmotic solution (0.735 g of sodium citrate dihydrate and 1.351 g of D-fructose in 100 mL distilled H_₂_O) and incubated at 37 °C for 30 min.

### 2.6 Evaluation of sperm chromatin condensation and DNA integrity

#### 2.6.1 Aniline blue (AB) staining

AB staining was done by following (25) with slight modifications. A 5 μL of semen was smeared on a microscopic glass slide and air-dried. Then, it was fixed with 2.5% glutaraldehyde in PBS for 30 min. Next, smears were stained with 5% aqueous aniline blue (pH 3.5) for 5 min and visualized under a bright-field microscope with 400× magnification. Sperm cells with good chromatin condensation remain unstained/light blue (AB⁻) in color, and poor chromatin condensation is stained blue (AB⁺) in color. A minimum of 200 cells per slide was counted.

#### 2.6.2 Sperm DNA fragmentation

The acridine orange (AO) fluorescence method was used to assess sperm DNA integrity by following the method described by (26) with slight modifications. A 100 µL of semen was centrifuged at 3,000 rpm for 10 min. The supernatant was discarded, and the pellet was mixed with PBS. A 200 μL of the mixture was then mixed with 20 μL of AO solution [containing 50 μg/mL purified AO (HIMEDIA)] and kept incubating for 5 min. A 5 μL of it was smeared on a microscopic glass slide and observed under a fluorescence microscope (excitation 450–490 nm) at 400x magnification. Sperm cells with intact DNA show green fluorescence, and those with denatured DNA exhibit red or orange fluorescence. A minimum of 200 sperm cells were counted.

### 2.7 Statistical analysis

Statistical analyses were performed in GraphPad Prism (Version: 10.6.1). Descriptive statistics were computed and summarized as mean and standard deviation (mean ± SD). A comparison between the groups (Control, Mild, Moderate, and Heavy) was made using a one-way analysis of variance, followed by Tukey's *post hoc* test for multiple pairwise comparisons. Multiple linear regression models, adjusting for baseline age and Body mass index (BMI), were utilized to assess the independent effects of alcohol consumption on seminal oxidative stress and nuclear damage. Correlations between alcohol consumption levels and semen parameters/oxidative stress markers were assessed using Pearson's correlation coefficient. A *p*-value of <0.05 was considered statistically significant.

## 3 Results

### 3.1 Demographic profile

The demographic profile of the study population is given in Table 1. A total of 126 individuals (aged between 20 and 45) who visited the Department of Reproductive Medicine at the SNHRC for fertility assessment and IVF predominantly belonged to the age group between 31 and 35 years old (42.06%), followed by 25–30 years old (34.12%), 36–40 years old (19.84%), and 41–45 years old (3.96%). BMI had revealed that approximately 43.65% of individuals were overweight, followed by normal weight range (34.12%), underweight (15.87%), and obese (6.34%). The occupational analysis indicated that half of the participants were unskilled workers, while a significant proportion were professionals (40.47%). Educational levels were diverse, with 30.95% of participants having completed primary education and 30.15% having completed secondary education, while 25.39% had attained tertiary education. Approximately 80.15% were reported consuming alcohol, which was consistent with the study's inclusion criteria, and 19.84% of people were non-alcoholics.

**Table 1:** Demographical characteristics of the study population.

Characteristics	Frequency	Percentage
Age (years)		
25–30	43	34.12
31–35	53	42.06
36–40	25	19.84
41–45	5	3.96
Body mass index (kg/m^2^)		
Underweight (≤18.5)	20	15.87
Normal weight (18.6–25)	43	34.12
Overweight (25–30)	55	43.65
Obese (≥30.1)	8	6.34
Education level		
Illiterate	17	13.49
Primary	39	30.95
Secondary	38	30.15
Tertiary	32	25.39
Occupation		
Professional	51	40.47
Unskilled	63	50
Unemployed	12	9.52
Alcohol consumption		
Yes	101	80.15
No	25	19.84

### 3.2 Effects of alcohol consumption on semen parameters

A significant dose-dependent deterioration in several key seminal parameters was observed with increasing alcohol consumption, summarized in Table 2. A statistically significant reduction was found in the semen volume of alcohol-consuming cohorts compared with the control cohort. The control group exhibited a mean sperm concentration of 195.12 ± 64.18 million/mL, whereas a decline was observed in the mild group (118.43 ± 46.91 million/mL), 72.64 ± 39.21 million/mL in the moderate group, and 32.60 ± 30.01 million/mL in the heavy group. A *post hoc* analysis confirmed that these differences were highly significant (*p* < 0.001) for all pairwise comparisons against the control group. Both total and progressive motility were severely compromised in the alcohol-consuming cohorts. The control group had a mean progressive motility of 47.68 ± 4.11%, whereas the mild group had 12.59 ± 3.32%, 9.35 ± 4.12% in the moderate group, and 5.96 ± 4.73% in the heavy group, which was aligning with their fertility issues. The control group maintained a total motility of 71.48 ± 5.19%. In contrast, the total motility was 45.70 ± 7.40% in the mild group, 37.29 ± 10.07% in the moderate group, and 15.78 ± 8.75% in the heavy group (*p* < 0.001).

**Table 2:** A comparison of standard semen parameters among different study groups. The table represents the mean and standard deviation of standard semen parameters.

Parameter	Control	Mild	Moderate	Heavy
Volume (mL)	3.46 ± 0.47	2.24 ± 0.56^a^	2.17 ± 0.55^a^	1.16 ± 0.31^a^
Sperm concentration (× million/mL)	195.12 ± 64.18	118.43 ± 46.91^a^	73.64 ± 39.21^a^	32.60 ± 30.01^a^
Progressive motility (%)	47.68 ± 4.11	12.59 ± 3.32^a^	9.35 ± 4.12^a^	5.96 ± 4.73^a^
Total motility (%)	71.48 ± 5.19	45.70 ± 7.40^a^	37.29 ± 10.07^a^	15.78 ± 8.75^a^
Normal morphology (%)	32.76 ± 8.19	2.21 ± 0.75^a^	1.87 ± 0.62^a^	1.09 ± 0.52^a^

a The statistically significant value of *p* < 0.001.

The major effect of the alcohol-associated oxidative stress was identified to be in sperm morphology, as all the alcohol-consuming cohorts exhibited teratozoospermia, which denotes that normal morphology is < 4%. The control group was found to have 32.76 ± 8.19% of cells with normal morphology. The percentage of morphologically normal sperm dropped drastically to 2.21 ± 0.75% in the mild group and 1.09 ± 0.52% in the heavy group. These values are well below the WHO (2021) lower reference limit of <4%, indicating that even long-term mild alcohol consumption is associated with a loss of structural integrity in sperm cells.

### 3.3 Impact of alcohol on seminal plasma composition and oxidative stress

The evaluation of accessory gland function and the extent of oxidative stress were assessed by quantifying seminal plasma biomarkers. Fructose is the major glycolytic energy substrate for spermatozoa, which is secreted primarily from the seminal vesicles. The levels of fructose exhibited a progressive decline based on the levels of alcohol consumption (Figure 2).

**Figure 2:**
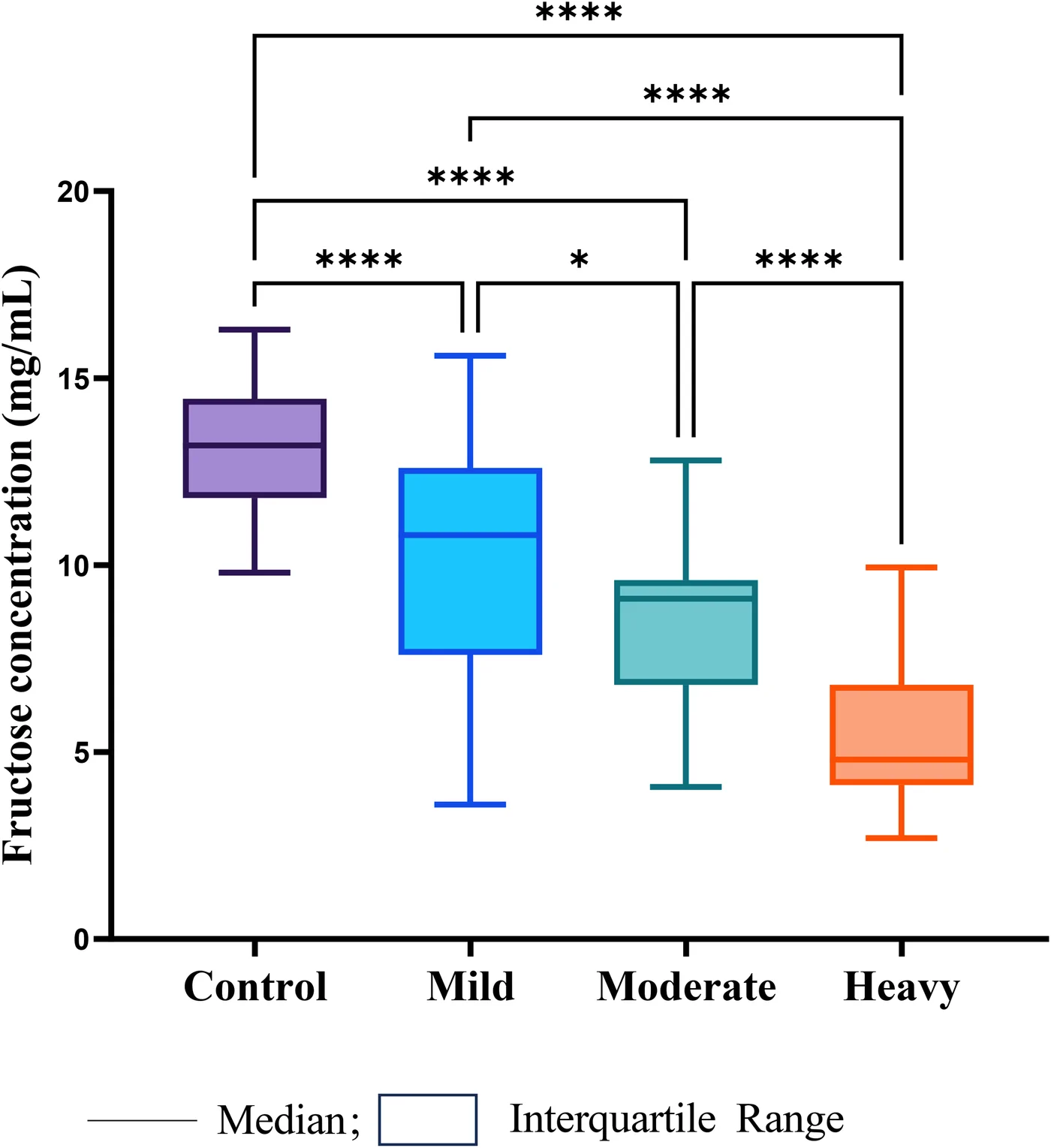
A box-and-whisker plot represents a comparison of fructose concentration (mg/mL) among the study cohorts. The whiskers represent the maximum and minimum values. **** indicates *p* < 0.0001 (control vs. mild/moderate/heavy; mild vs. heavy; moderate vs. heavy). * indicates *p* < 0.05 (mild vs. moderate). A comparison of fructose concentration among the study cohorts.

The control group had a mean fructose concentration of 13.07 ± 1.64 mg/mL, whereas the mild group had 10.2 ± 3.15 mg/mL, 8.42 ± 1.91 mg/mL in the moderate group, and 5.45 ± 1.94 mg/mL in the heavy group (*p* < 0.0001). A *post hoc* analysis confirmed that even mild alcohol consumption reduced fructose levels significantly compared with controls (*p* < 0.0001). The decline in fructose levels was aligned with the levels of alcohol intake; the moderate group exhibited significantly lower levels than the mild group (*p* < 0.05), and the heavy group was found to have the lowest concentration, significantly differing from both the moderate and the mild groups (*p* < 0.0001).

A linear reduction in TPC was observed in the alcohol-consuming cohorts (Figure 3). The control group had a mean TPC of 44.24 ± 3.82 mg/mL, whereas the mild group had 35.03 ± 3.54 mg/mL, 28.79 ± 4.43 mg/mL in the moderate group, and 21.8 ± 5.22 mg/mL in the heavy group (*p* < 0.0001). Unlike fructose, the statistical difference between the mild and moderate groups for protein was highly significant (*p* < 0.0001), showing that prostatic secretory function may be highly sensitive to increasing alcohol doses.

**Figure 3:**
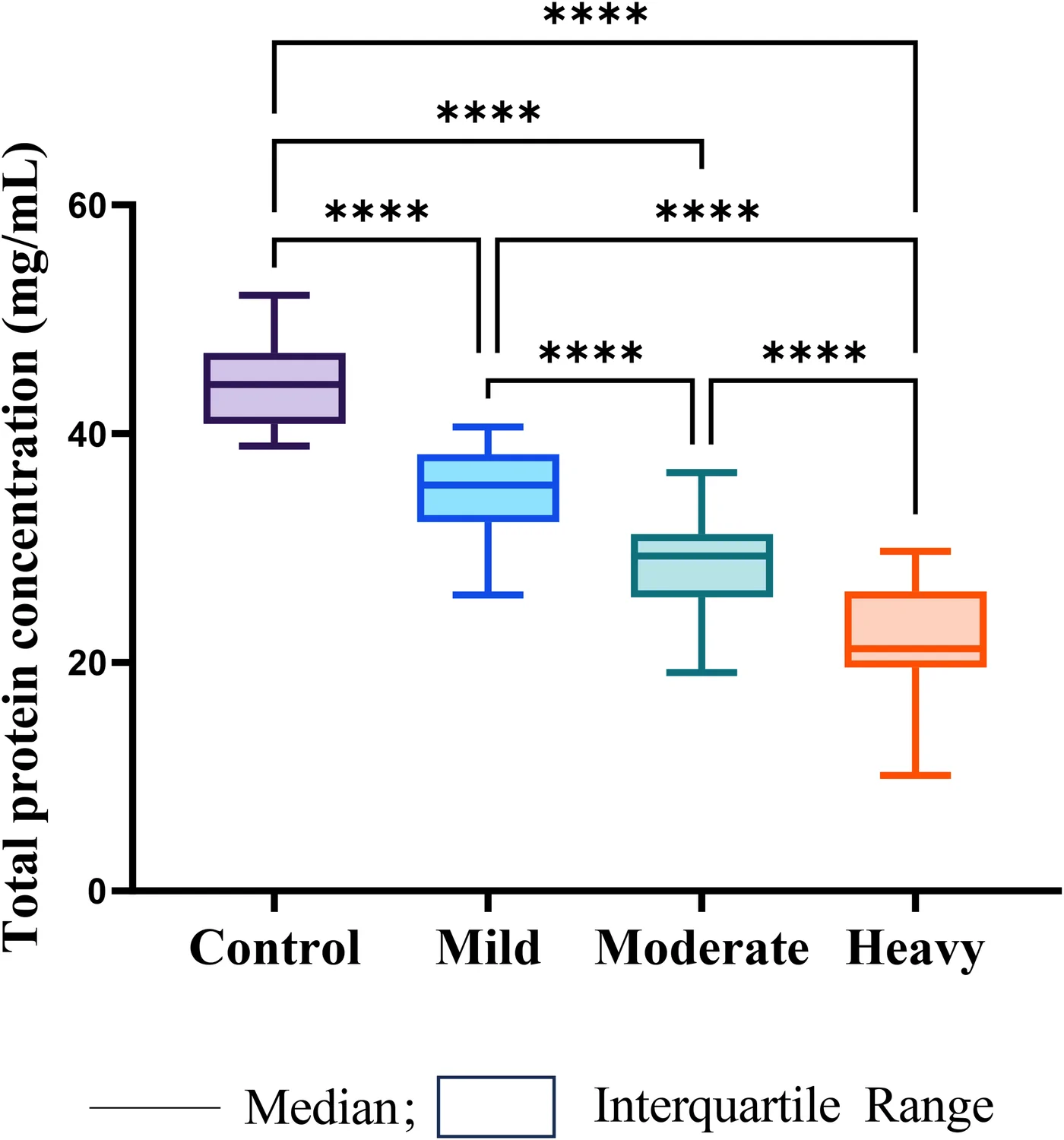
A box-and-whisker plot showing a comparison of TPC among the study cohorts. The whiskers represent the maximum and minimum values. A highly significant decrease was observed across all groups. **** indicates *p* < 0.0001 for all pairwise comparisons (control vs. mild/moderate/heavy; mild vs. moderate/heavy; moderate vs. heavy). A comparison of TPC among the study cohorts.

Lipid peroxidation levels were concomitant with the decline in protective seminal fluids; a profound elevation in oxidative stress was observed (Figure 4). Seminal MDA levels, a marker of oxidative lipid degradation, increased significantly with alcohol intake. The mild group exhibited a statistically significant rise in MDA compared with controls (*p* < 0.0001). The oxidative burden intensified progressively along with the levels of alcohol consumption. The moderate group showed significantly higher MDA levels than the mild group (*p* < 0.001), and the heavy group displayed the highest median levels, significantly exceeding the moderate group (*p* < 0.0001).

**Figure 4:**
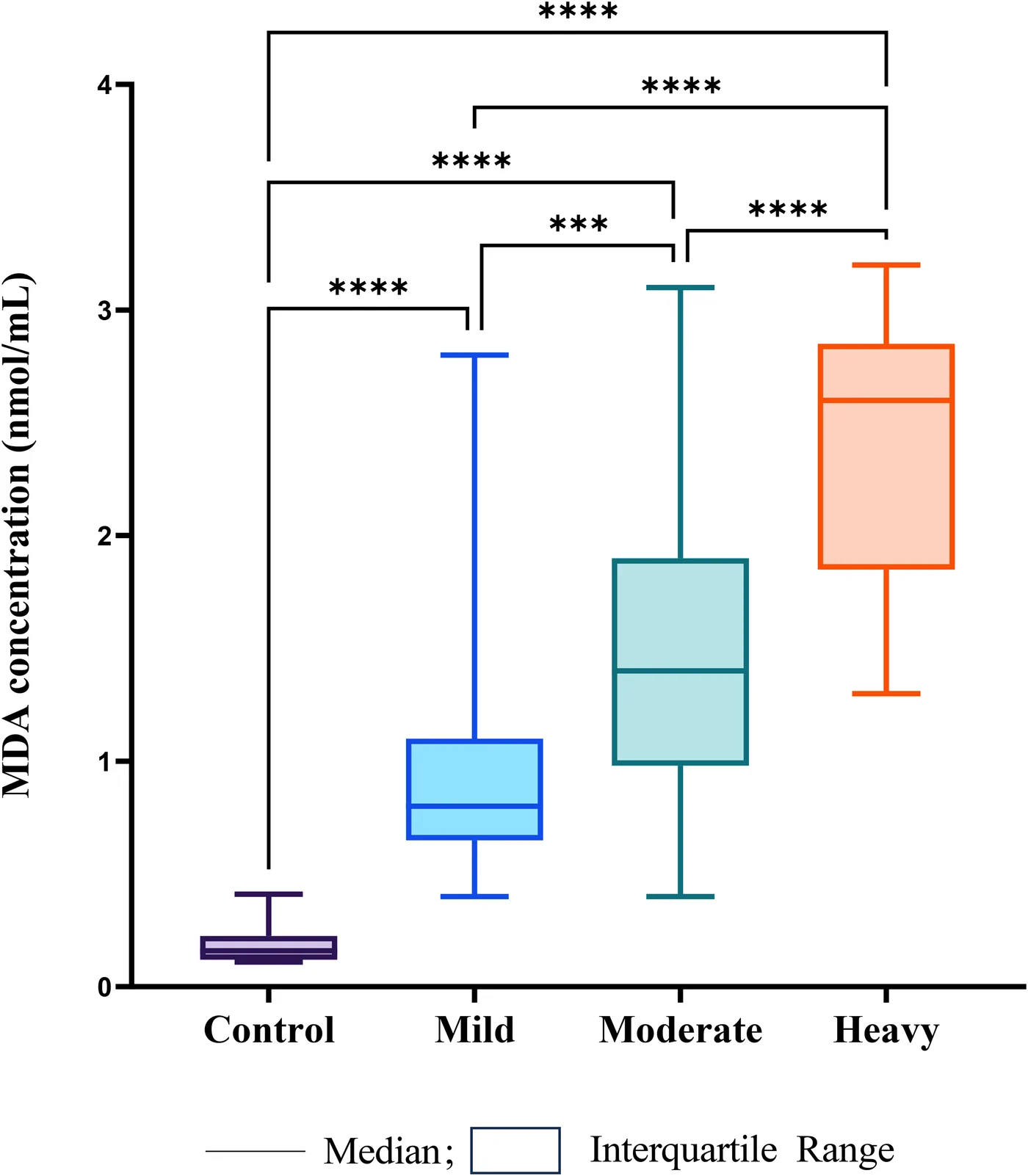
A box-and-whisker plot represents a comparison of seminal MDA levels (nmol/mL) among the study cohorts. The whiskers represent the maximum and minimum values. A significant increase in oxidative stress was observed among the alcohol-consuming cohorts. **** indicates *p* < 0.0001 (control vs. mild/moderate/heavy; moderate vs. heavy). *** indicates *p* < 0.001 (mild vs. moderate). A comparison of MDA concentration among the study cohorts.

### 3.4 Scanning electron microscopy analysis

Following the identification of decline in sperm morphology via light microscopy across all study cohorts (*n* = 126), an SEM analysis was utilized purely as a qualitative visual confirmation of structural defects rather than quantitative diagnostic morphology scoring. The SEM focused on the heavy group to provide the clearest visual evidence of ultrastructural damage compared with the control. Figure 5 represents the SEM images illustrating sperm morphology. (A) represents the SEM image of the control group, and (B) represents the SEM image of the heavy alcoholic group.

**Figure 5:**
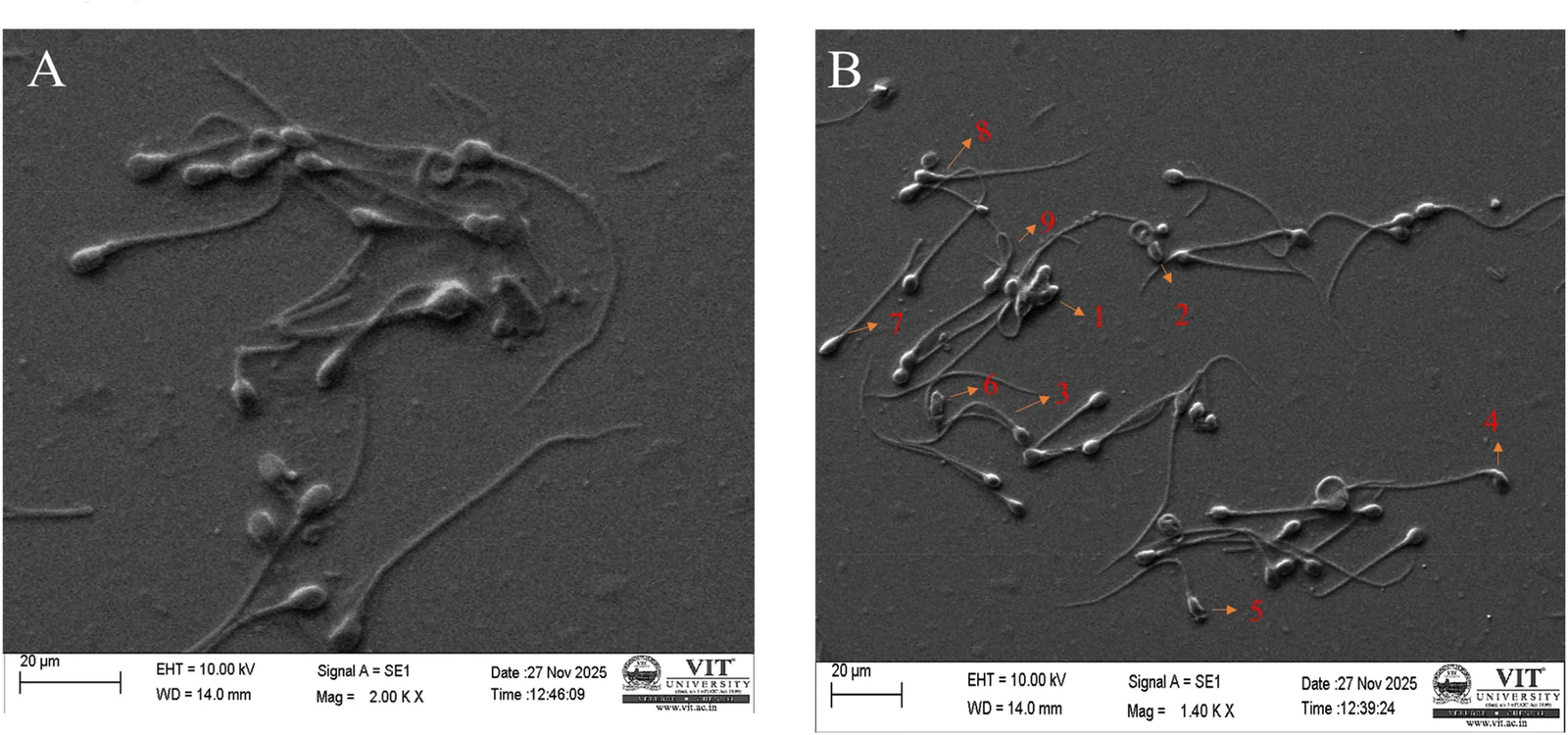
**(A)** An SEM image shows that most of the sperm cells in the control group resemble normal morphology. **(B)** The SEM image represents the extremes of morphological divergence in the sperm population of the heavy group. 1, Multiple heads; 2, coiled tail; 3, double tail; 4, bent neck; 5 and 6, amorphous/irregular head; 7, thin elongated head and a short tail; 8, immature sperm cell; and 9, amorphous head with a bent tail.

### 3.5 Sperm membrane integrity

Sperm cells with an intact plasma membrane undergo fluid influx to maintain osmotic equilibrium. This results in the coiling of the sperm tail, indicating a positive hypo-osmotic swelling (HOS) test result (Figure 6). According to WHO criteria, a HOS test score above 55% is considered indicative of normal membrane integrity and healthy reproductive potential. In response to hypo-osmotic conditions, the control group demonstrated a mean of 66.9 ± 4.4% of sperm cells with coiled and swollen tails. The alcohol-consuming cohorts exhibited lower mean HOS positive rates: 42.1 ± 10.1% in the mild group, 40 ± 11.8% in the moderate group, and 37.7 ± 9.5% in the heavy group. This demonstrates the reduction in sperm membrane integrity corresponding to increased alcohol consumption.

**Figure 6:**
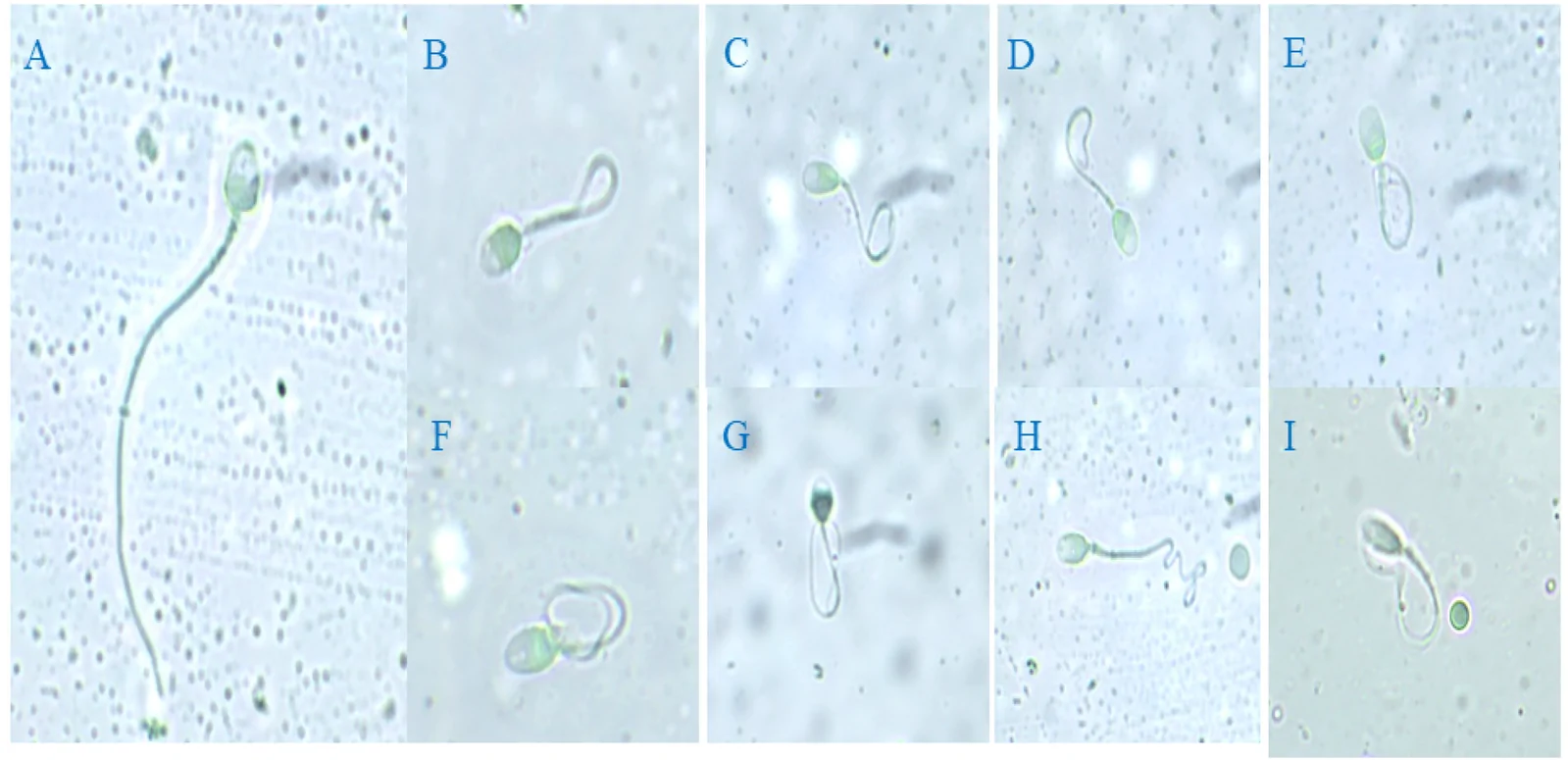
(**A)** HOS negative, **(B–I)** HOS positive. The images represent the response of sperm cells to a hypo-osmotic swelling test observed in 400× magnification under a bright-field microscope.

### 3.6 Evaluation of chromatin condensation and sperm DNA integrity

The impact of alcohol on sperm nuclear maturity was assessed using AB staining to quantify histone retention. Figure 7 represents the results of AB staining in sperm cells; AB^+^ indicates sperm cells with poor chromatin condensation, which is aniline blue positive, and the unstained cells were considered to have proper chromatin condensation.

**Figure 7:**
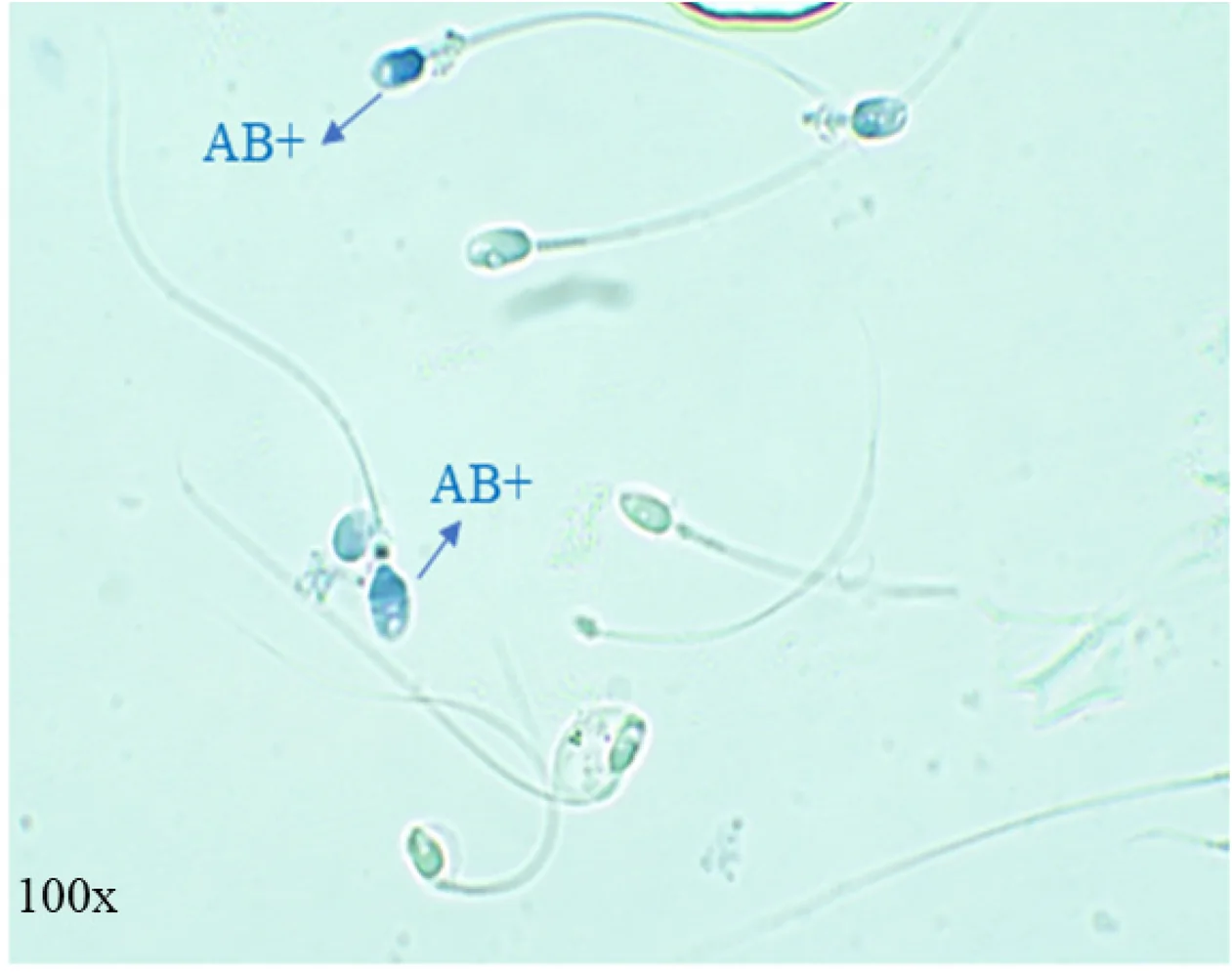
Light Micrograph of Human Sperm cells stained with Aniline Blue. AB+ indicates sperm cells with poor chromatin condensation with high histone persistence. Other sperm cells without stain indicate sperm cells with tightly packed chromatin.

The distribution of chromatin defects across the study groups is presented as a box-and-whisker plot in Figure 8. The control group exhibited a low mean percentage of aniline blue-positive (AB^+^) spermatozoa, 13.6 ± 3.07%, with a narrow standard deviation reflecting consistent nuclear maturity in the control group. Alcohol consumption was found to be associated with poor chromatin condensation. The mild group showed a statistically significant elevation in the mean AB^+^ rate of 40.2 ± 9.8% compared with the control group (*p* < 0.0001), indicating that even long-term mild alcohol consumption may impact histone-to-protamine transition. The heavy group displayed the highest mean rate of histone retention with 56.2 ± 8.6%, which was significantly higher than both the moderate (*p* < 0.01) and mild (*p* < 0.0001) groups. The wider standard deviations observed in the alcohol-consuming cohorts suggest increased interindividual variability in susceptibility to alcohol-induced chromatin damage.

**Figure 8:**
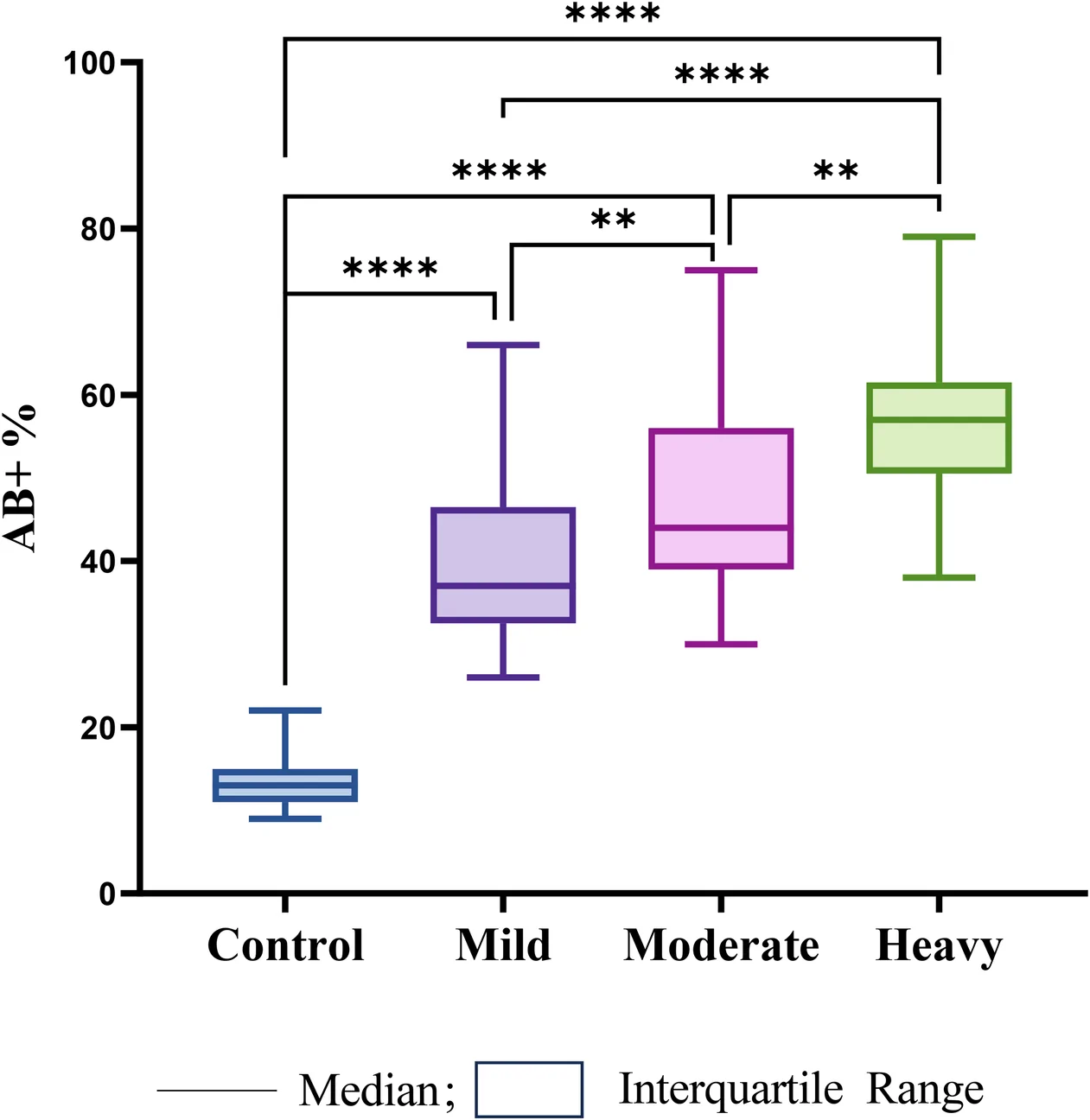
A box-and-whisker plot representing the distribution and comparison of the percentage of AB^+^ sperm cells across the study cohorts. The whiskers represent the minimum and maximum values. **** indicates *p* < 0.0001 (control vs. mild/moderate/heavy; mild vs. heavy). ** indicates *p* < 0.01 (mild vs. moderate; moderate vs. heavy). A comparison of sperm chromatin condensation among the study cohorts.

An analysis of sperm DNA integrity using the acridine orange fluorescence assay revealed a similar pattern (Figure 9). The control group maintained a low baseline mean DNA fragmentation index (DFI) of 7.7 ± 1.7%, indicating stable chromatin. In contrast, long-term alcohol consumption at any level was associated with a significant loss of DNA integrity in alcohol-consuming cohorts.

**Figure 9:**
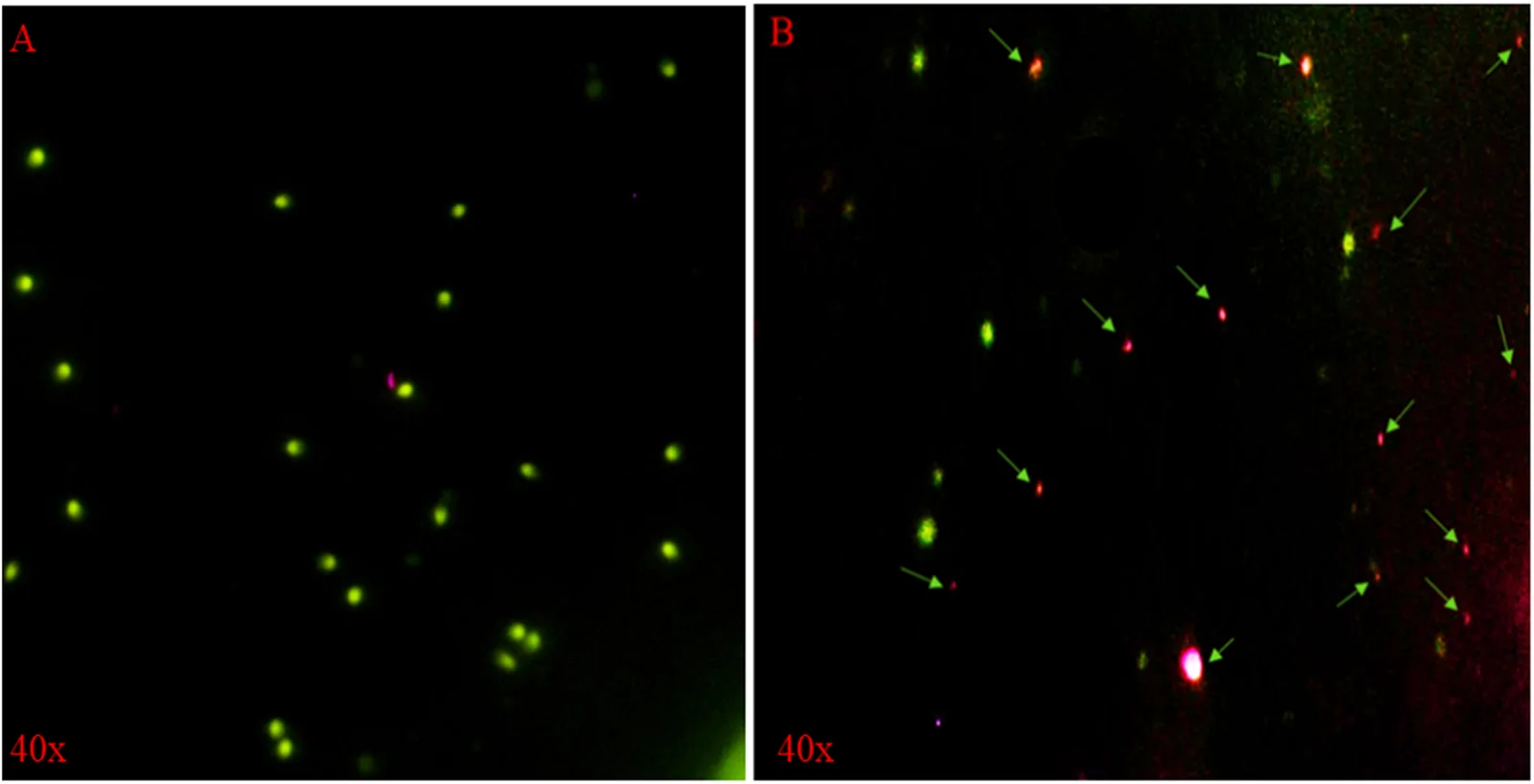
Representative fluorescence microscopy images of sperm cells evaluated for DNA fragmentation using AO staining. **(A)** Green fluorescence indicates normal sperm cells with intact double-stranded DNA. **(B)** Red or orange fluorescence indicates sperm cells with denatured or fragmented single-stranded DNA (green arrows).

The mild group exhibited a significantly higher mean DFI of 22.4 ± 9.6% compared with the control group (*p* < 0.0001). The severity of DNA damage progressed with increasing alcohol intake, as the heavy group demonstrated the highest mean DFI of 37.2 ± 8.9% and the widest standard deviation, significantly exceeding both the moderate (28.5 ± 7.7%, *p* < 0.001) and mild (*p* < 0.0001) groups. A statistically significant difference was also observed between the mild and moderate groups (*p* < 0.05), confirming that DNA damage increases progressively with alcohol consumption levels (Figure 10).

**Figure 10:**
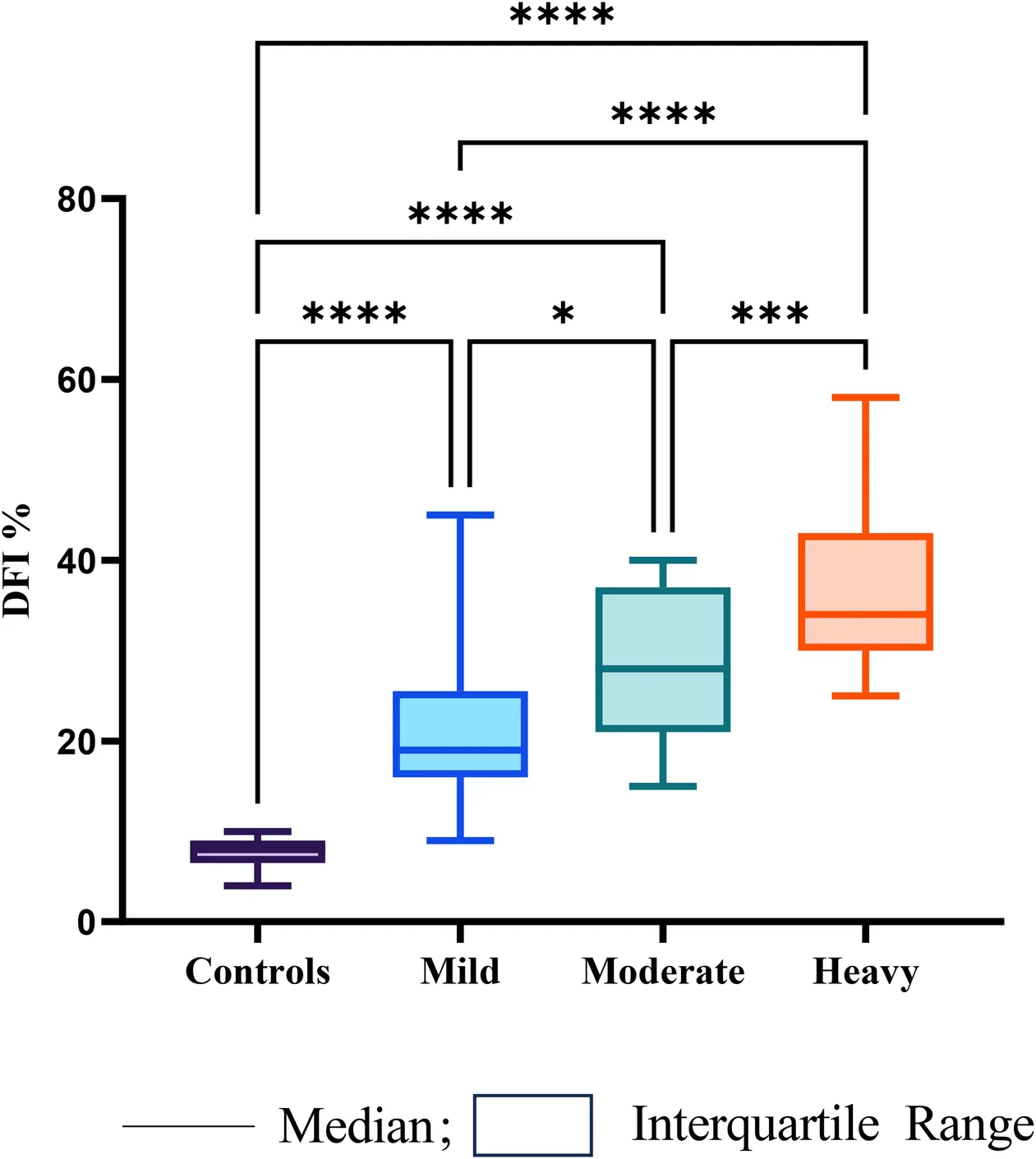
A box-and-whisker plot illustrating the distribution and comparison of sperm DFI across the study cohorts. The whiskers represent the minimum and maximum values. **** indicates *p* < 0.0001 (control vs. mild/moderate/heavy; mild vs. heavy). *** indicates *p* < 0.001 (moderate vs. heavy). * Indicates *p* < 0.05 (mild vs. moderate). A comparison of DNA damage among the study cohorts.

### 3.7 Adjusted analysis for confounding variables

To confirm the independent effects of alcohol consumption, the data were adjusted for baseline demographic confounders, specifically age and BMI. Multiple linear regression models confirmed that even after adjusting for these two variables, alcohol remained a highly significant driver of elevated oxidative stress and genomic instability. The adjusted values of seminal oxidative stress and DNA integrity markers are presented in Table 3. Specifically, an analysis revealed that alcohol exposure was the independent predictor of both elevated sperm DNA fragmentation index (*p* < 0.0001) and seminal MDA concentrations (*p* < 0.0001). Although advancing paternal age was identified as a minor contributor for both DFI (*p* = 0.0265) and oxidative stress (*p* = 0.0340), BMI showed no significant impact (*p* = 0.3931), underscoring alcohol as the primary driver of these pathologies.

**Table 3:** A multiple linear regression analysis of variables associated with seminal oxidative stress and nuclear damage.

Dependent variable	Predictor variable	Adjusted values (*β*)	95% CI	*P*-values
MDA (nmol/mL)	Mild	0.760	0.492–1.028	˂0.0001
	Moderate	1.246	0.966–1.525	˂0.0001
	Heavy	2.192	1.917–2.466	˂0.0001
	Age	0.027	0.002–0.051	0.0340
	BMI	0.001	−0.021–0.024	NS
DFI (%)	Mild	14.86	10.83–18.88	˂0.0001
	Moderate	20.69	16.49–24.89	˂0.0001
	Heavy	29.45	25.32–33.57	˂0.0001
	Age	0.422	0.050–0.795	0.0265
	BMI	−0.149	−0.493–0.195	NS

NS, not significant. Adjusted values (*β*) represent the estimated mean difference relative to the control group.

## 4 Correlation between oxidative stress markers and sperm quality

To evaluate the mechanism underlying alcohol-associated male infertility, Pearson's correlation analysis was performed between seminal oxidative stress markers, accessory gland secretory products, and sperm quality parameters. Pearson's coefficients were calculated using the cumulative data of the entire study population (*n* = 126) (Table 4), encompassing both the control and the alcohol-consuming cohorts.

**Table 4:** The Pearson's correlation coefficients (*r*) between seminal oxidative stress markers and sperm quality parameters across the entire study population (*n* = 126).

Semen quality parameters	MDA concentration (nmol/mL)	Fructose (mg/mL)	Total protein concentration (mg/mL)
Volume	−0.644^a^	0.631^a^	0.673^a^
Sperm concentration	−0.631^a^	0.706^a^	0.719^a^
Progressive motility	−0.687^a^	0.709^a^	0.762^a^
Total motility	−0.811^a^	0.867^a^	0.843^a^
Normal morphology	−0.595^a^	0.580^a^	0.703^a^
Chromatin condensation (AB)	0.841^a^	−0.646^a^	−0.753^a^
DNA fragmentation index (DFI)	0.880^a^	−0.656^a^	−0.755^a^

Values represent Pearson’s correlation coefficient (*r*). All correlations were statistically significant.

a *p* < 0.0001.

A strong positive correlation was observed between lipid peroxidation (MDA concentration) and markers of nuclear and chromatin instability (DFI and AB^+^). Conversely, MDA exhibited strong negative correlations with vital seminal constituents like fructose, TPC, and overall sperm motility. A significant strong positive correlation between seminal MDA levels and sperm DFI was observed (r = 0.880, *p* < 0.0001), indicating that alcohol-associated oxidative stress may be the primary driver of genomic instability in the sperm cells. Beyond the genomic damage, a significant positive correlation was observed between seminal fructose concentration and total motility (r = 0.867, *p* < 0.0001). Because fructose serves as the primary glycolytic substrate for sperm metabolism, the alcohol-associated secretory dysfunction leads to depletion in the energy source required for sperm cell movement. Furthermore, TPC exhibited a strong inverse relationship with the DFI (r = −0.755, *p* < 0.0001) and chromatin condensation defects (r = −0.753, *p* < 0.0001).

The profound negative correlation highlights the protective role of seminal plasma proteins, which include various antioxidant enzymes and decapacitation factors. The depletion of these proteins leaves the sperm nuclei more vulnerable to external oxidative stress, which is evidenced by the elevated MDA levels. It was found that these correlations reveal a multifaceted decline in male reproductive health as alcohol consumption increases; the rise in oxidative stress works in tandem with the depletion of essential seminal components like fructose and TPC to drive a catastrophic loss of both sperm motility and nuclear integrity. The heatmap represents the unadjusted Pearson correlation coefficients (r) across the entire study population (*n* = 126) (Figure 11). Numerical values and color intensity represent the strength and direction of the linear relationships, with positive associations depicted in blue and negative associations in red.

**Figure 11:**
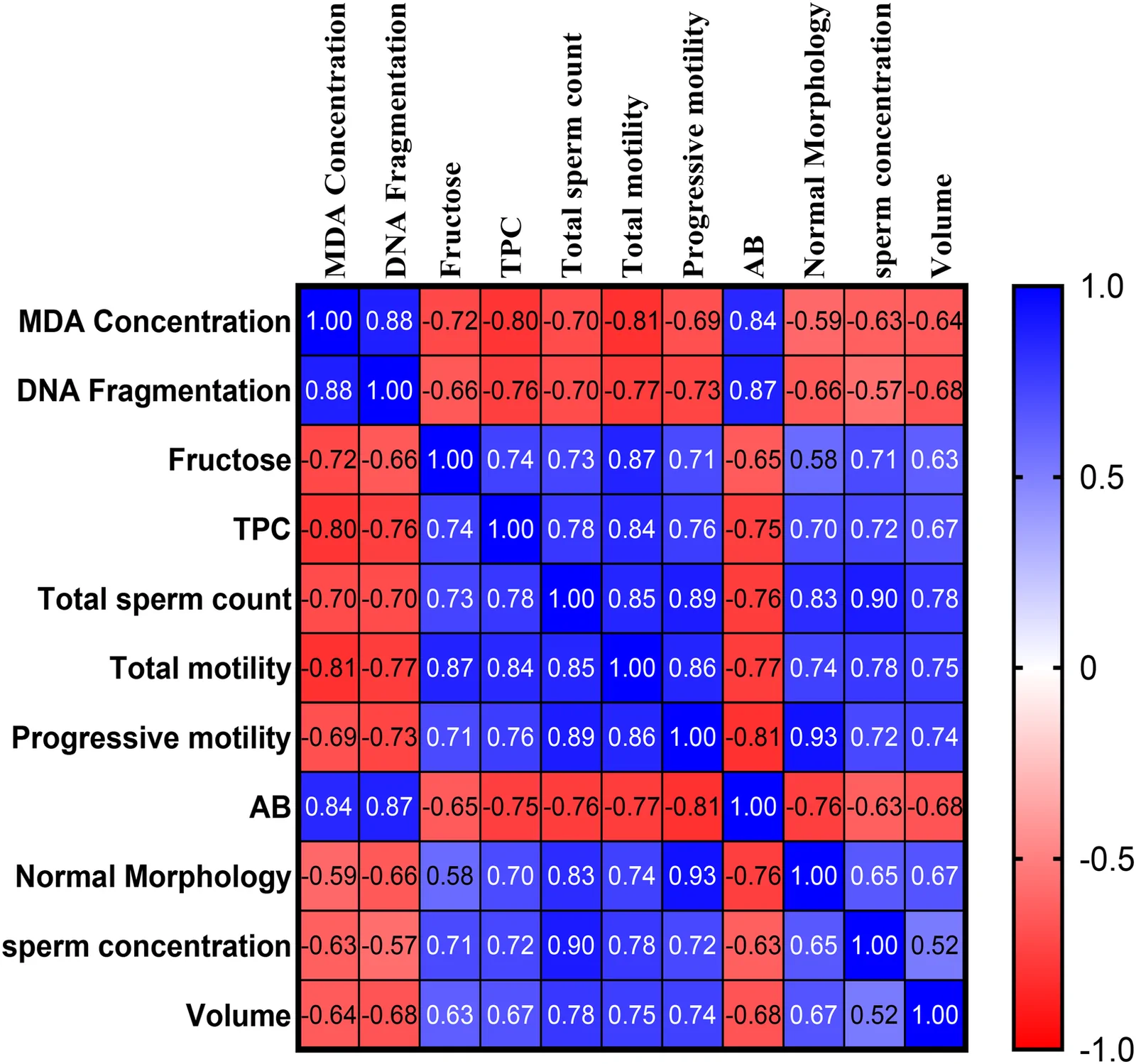
A heatmap demonstrating the relationships between seminal oxidative stress, accessory gland secretions, and sperm quality parameters.

## 5 Discussion

Our study provides a comprehensive evaluation of the effects of alcohol consumption on male reproductive health. It was found that long-term alcohol consumption at any level affects semen quality by reducing accessory gland secretion and increasing oxidative stress–mediated genomic instability.

The observed impacts on semen quality parameters across all alcohol-consuming cohorts compared with the control group align with the recent meta-analysis. For instance, Nguyen-Thanh et al. confirmed a significant inverse correlation between daily alcohol intake and standard semen parameters like sperm concentration and morphology (12). Finelli et al. highlighted that poor semen quality was associated with chronic alcohol consumption due to oxidative stress and genomic instability (11). However, these previous meta-analyses primarily focused on the standard WHO endpoints, our findings expand on this clinical foundation by demonstrating that the observed deterioration of semen quality is mechanically linked to seminal plasma oxidative stress and the depletion of protective accessory gland secretions. Notably, even long-term mild alcohol consumption in infertile men was found to have compromised semen quality, challenging the notion that low-level intake is benign, aligning with the recent findings demonstrating that modifiable lifestyle factors exert a synergistic and cumulative toxic burden on spermatogenesis (5).

The profound decline in normal morphology suggests that alcohol disrupts cytoskeletal organization during spermiogenesis, which is consistent with the recent findings (13). Notably, an SEM analysis revealed that significant alcohol consumption was most frequently associated with head anomalies (particularly amorphous heads) and cytoskeletal defects (such as coiled tails) (Figure 5B). The prevalence of coiled tails specifically corroborates the HOS test findings, suggesting that alcohol-associated lipid peroxidation severely compromises the flagellar plasma membrane. Furthermore, a decline in the seminal fructose and TPC was observed in alcohol-consuming cohorts. Because fructose is a major glycolytic metabolite for sperm motility, the observed strong positive correlation between fructose and total motility (r = 0.867, *p* < 0.0001) suggests that the decreased motility is driven by reduced secretory capacity of the accessory glands.

Particularly, the elevated MDA levels observed in the heavy group may be due to the alcohol metabolism via the CYP2E1 pathway, which is known to generate excessive ROS (18)*.* The observed strong negative correlation between MDA and total motility (r = −0.811) confirms the peroxidative damage to the sperm cell membrane, which compromises membrane fluidity and flagellar motion. The observed very strong positive correlation between MDA and sperm DFI (r = 0.880, *p* < 0.0001) confirms that oxidative stress is a primary inducer of genotoxicity.

Furthermore, the disruption in the levels of histone retention observed in the aniline blue staining in the alcohol-consuming cohorts indicates the impact of alcohol on histone-to-protamine transition during the elongation phase of spermiogenesis, which results in immature chromatin (19)*.* 4-HNE, which is a by-product of lipid peroxidation, may form covalent adducts with cystine-rich protamine. This biochemical interference can possibly hamper inter- and intramolecular disulfide bridges, preventing the tight chromatin packaging crucial for genomic integrity in sperm cells. This results in the deterioration of the sperm nucleus, which makes it physically unstable and highly susceptible to fragmentation. Research studies highlight that sperm DNA fragmentation is not only a barrier for fertilization but also a determinant of embryo health, as sperm with fragmented DNA may fertilize an oocyte but often lead to implantation failure or early recurrent pregnancy loss and affect the blastocyst formation rate (27)*.*

Collectively, the observed decline in the semen quality of the alcohol-consuming cohorts is consistent with a broader reconsideration of the notion of a “moderate” or “safe” threshold of alcohol intake in the context of male reproductive health. The findings align with the position articulated by the WHO, which states that no level of alcohol consumption can be considered entirely free of health risk (28)*.* Unlike genetic determinants of semen quality, which are largely non-modifiable, lifestyle factors are modifiable and therefore constitute a practical, actionable target for improving reproductive outcomes in men undergoing fertility evaluation.

## 6 Conclusion

Alcohol consumption affects male reproductive health through multiple interrelated mechanisms. In this study, it was found that it was associated with reduced secretory capacity of the accessory glands, leading to suppression of the energy metabolites required for sperm motility. Alcohol consumption was also associated with systemic oxidative stress that damages the sperm cell membrane and disrupts chromatin packaging, ultimately contributing to poor DNA integrity. Sperm DNA fragmentation appeared to be a more sensitive indicator of alcohol-associated changes than standard semen parameters. These findings support alcohol reduction as a modifiable lifestyle factor worth addressing in fertility counseling, particularly for men undergoing assisted reproductive techniques. However, prospective multicenter studies are needed before specific clinical abstinence recommendations can be justified.

### 6.1 Limitations

Although the findings of this study provide comprehensive insights into alcohol-associated reproductive toxicity, some limitations must be acknowledged.

Although the total cohort size (*n* = 126) provided adequate statistical power, stratifying the participants into four distinct subgroups resulted in relatively small sample sizes per group. This restricted sample size was an unavoidable consequence of our rigorous exclusion criteria, as isolating alcohol consumption as an independent variable requires finding subjects who do not engage in confounding lifestyle habits such as smoking or recreational drug use.

Finally, because this research was conducted on a specific cohort of men undergoing clinical fertility evaluation, extrapolating these findings to the broader, general male population should be done with caution. This focused cohort was selected to uncover the precise biochemical pathology in men currently facing reproductive challenges, thereby maximizing the immediate translational value of these findings for clinical andrology and assisted reproductive technologies.

## Data Availability

The original contributions presented in the study are included in the article/Supplementary Material; further inquiries can be directed to the corresponding author.
